# Barriers and facilitators to paediatric adherence to antiretroviral therapy in rural South Africa: a multi-stakeholder perspective

**DOI:** 10.1080/09540121.2014.967658

**Published:** 2014-10-30

**Authors:** Bronwyne Coetzee, Ashraf Kagee, Ruth Bland

**Affiliations:** ^a^ Department of Psychology, Stellenbosch University, Stellenbosch, South Africa; ^b^ Africa Centre for Health and Population Studies, KwaZulu-Natal, South Africa; ^c^ Royal Hospital for Sick Children, University of Glasgow, Glasgow, UK; ^d^ School of Public Health, Faculty of Health Sciences, University of Witwatersrand, Johannesburg, South Africa

**Keywords:** HIV, adherence, barriers, facilitators, paediatric, anti-retroviral treatment

## Abstract

Poor adherence to antiretroviral therapy (ART) contributes to the development of drug resistance. HIV-infected children, especially those 5 years and under, are dependent on a caregiver to adhere to ART. However, characteristics of the caregiver, child, regimen, clinic and social context affect clinic attendance and medication-taking, both of which constitute adherent behaviour. We conducted nine interviews and three focus groups to determine how doctors, nurses, counsellors, traditional healers and caregivers understood the barriers and facilitators to ART adherence among children residing in rural South Africa. The data were transcribed, translated into English from isiZulu where necessary, and coded using Atlas.ti version 7. Results were interpreted through the lens of Bronfenbrenner's Ecological Systems Theory. We found that at the micro-level, palatability of medication and large volumes of medication were problematic for young children. Characteristics of the caregiver including absent mothers, grandmothers as caregivers and denial of HIV amongst fathers were themes related to the micro-system. Language barriers and inconsistent attendance of caregivers to monthly clinic visits were factors affecting adherence in the meso-system. Adherence counselling and training were the most problematic features in the exo-system. In the macro-system, the effects of food insecurity and the controversy surrounding the use of traditional medicines were most salient. Increased supervision and regular training amongst lay adherence counsellors are needed, as well as regular monitoring of the persons attending the clinic on the child's behalf.

## Introduction

Excellent adherence (>95%) to antiretroviral therapy (ART) is required to achieve optimal outcomes of HIV treatment (Bangsberg et al., 2001). Paediatric adherence to ART is complicated by several factors associated with the caregiver, child and regimen (Haberer & Mellins, 2009).

Poor adherence may result in an unsuppressed viral load, leading to opportunistic infections, drug resistance and ultimately mortality (Arrivillaga, Martucci, Hoyos, & Arango, 2013). With limited ART drug options available in South Africa (Davies et al., 2011), adherence to first-line medication is key to ensuring optimal and prolonged treatment benefits.

**Figure 1.**
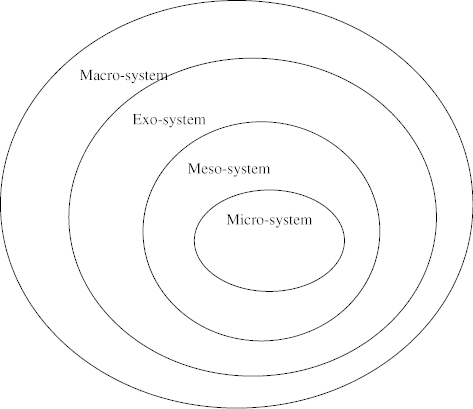
The four systems of Bronfenbrenner's EST.

Bronfenbrenner's (1979) Ecological Systems Theory (EST) was a suitable lens through which to design the study and interpret the findings. Bronfenbrenner describes four nested levels of influence, namely, micro-, meso-, exo- and macro-systems, which may be used to explain how a child's development and growth are affected by his or her environment (Figure 1). In our study, and specifically pertaining to a young child on ART, the child is situated in the micro-system alongside all those directly involved in his/her treatment and care. The meso-system involves the interactions between the members of the micro-system (e.g., the interaction between caregivers and counsellors), the exo-system considers the effect of the health care system, and the macro-system is concerned with the impact of culture, socio-economic status and poverty on adherence to ART. The present study sought to identify the barriers and facilitators to paediatric adherence to ART in a rural area in South Africa.

## Methods

### Setting

The study was conducted at the Africa Centre for Health and Population Studies (AC) South Africa (SA) (www.africacentre.ac.za) and a nearby peri-urban Department of Health (DoH) clinic. The AC has supported the DoH Hlabisa HIV Treatment and Care Programme (ART programme) since its inception in 2004 (Houlihan et al., 2011; Janssen, Ndirangu, Newell, & Bland, 2010).

#### Participants and procedure

Semi-structured in-depth interviews (IDIs) and focus groups (FGs) were conducted with purposively sampled individuals:

1. Doctors (IDI), nurses (IDI) and counsellors (FG) working within the ART programme;
2. Caregivers (FG) of children ≤5 years attending a peri-urban clinic;
3. Traditional healers (FG) consulting to people with HIV.

Using open-ended questions we explored participants' understanding of the barriers and facilitators to ART adherence amongst children. Interviews and FGs were conducted under confidential conditions in the participants' first language (isiZulu) with the assistance of an isiZulu speaking translator, or otherwise in English, either at the AC, clinic or hospital.

Examples of the questions used are shown in Table 1.

**Table 1.** Examples of the interview and FG question schedule.

FG schedule: Traditional healers
What do you see as the most important factors that act as barriers to caregivers when they provide children with their medications?
What do you see as the most important factors that act as facilitators to caregivers when they provide children with their medications?
Do you generally think that caregivers provide medications in the way that they are supposed to?
Why do you think they might not be providing the medication in the correct ways?
FG schedule: Caregivers
Please tell me about your experience with giving medicine to a child as their caregiver.
How would you describe your relationship with the child?
What difficulties do you think the child has in taking their medications?
What are the main problems that you have when administering medication to the child?
Interview guide: Health care providers (nurses and doctors) and HIV counsellor FG
Please tell me about your experience in treating children with HIV.
What do you think is the hardest part for children in taking their medication?
What is your experience of the role of caregivers in paediatric adherence?
Probe: interactions at clinic and home (do children listen to them; take medication when supposed to?)
Following regimen at home?
Knowledge of regimen and health literacy (do they struggle to understand how to administer the medication?)

Ethical approval was obtained from Stellenbosch University, with reciprocity from the University of KwaZulu-Natal.

### Data analysis

Interviews and FGs were recorded, transcribed, translated where necessary into English (and back translated) and entered into the Atlas.ti version 7 (www.atlasti.com) computer program (Friese, 2012). Data were analysed according to inductive thematic analytic procedures. We systematically familiarised ourselves with the data, coded relevant text segments, organised codes into categories and developed categories into latent themes and sub themes (Braun & Clarke, 2006). Bronfenbrenner's EST was used to organise latent themes at the individual, micro-, meso-, exo and macro levels.

## Results

### Respondent characteristics

We obtained data from 5 doctors (3 females, 2 males), 4 nurses (female), 12 counsellors (11 females, 1 male), 10 traditional healers (7 males, 3 females) and 11 caregivers (female). Table 2 summarises information from the caregivers in the sample.

**Table 2.** Characteristics of caregivers (*n* = 11).

Age of caregiver		
Mean (SD) years	32.9 (16.3)	
Caregivers relation to child	*N* (*N* = 11)	Per cent
Mother	7	63.6
Grandmother	3	27.3
Relative	1	9.1
Gender of child		
Male	3	27.3
Female	8	72.7
Caregiver marital status		
Single	8	72.7
Married or living in significant marriage-like relationship	2	18.2
Widowed	1	9.1
Highest level of education of caregiver		
No formal education	1	9.1
Attended high school but did not complete Grade 12^a^	7	63.6
Completed Grade 12	2	18.2
Graduated from university, college or technikon	1	9.1
Employment		
Unemployed	11	100
Income		
No income	1	9.1
Less than R12000 per month	3	27.3
More than R12000 per month	1	9.1
Grant R280 per month	4	36.4
Grant more than R280 per month	2	18.2
Received paediatric pre-ART HIV education		
Yes	9	81.8
No	2	18.2
Viral load (VL) status of child		
Suppressed^b^	6	54.5
Unsuppressed^c^	2	18.2
New enroller^d^	3	27.3

^a^ Grade 12 is the final year of secondary school; ^b^VL < 400cps/ml after 12 months on ART; ^c^VL > 400 cps/ml less than 12 months on ART; ^d^On ART < 6months- not yet received first VL.

Table 3 summarises the results below and contains selected quotations from the interviews and FGs.

**Table 3.** Themes and sub-themes identified through thematic analytic techniques.

			Interviews (selected quotations)		FGs (selected quotations)		
System	Description	Themes and *sub-themes*	Doctor	Nurse	Counsellor	Traditional healer	Caregiver
Individual	This system involves characteristics specific to the child that influences adherence to ART.	Medication difficulties *Palatability and side effects Large volumes of medication and swallowing tablets Blood tests*	The children are certainly nervous because they have often had blood tests done and the thought of coming to clinic is often quite worrying for them [Doctor 2].	So that's the, problematic one because they can say children will drink all the other medication but when it's this one they can even identify the bottle and then decide to close the mouth and even when they force them they spit [Nurse 2].	Sometimes they come in there, they're just feeling they don't feel like taking the medication. They can take it and they're doing like vomiting the thing. They spit the thing and you can't see that they didn't swallow that thing and that's a problem [Counsellor 4].	One lady mentioned that the child was refusing to drink the medication so she just opened the capsules and diluted them and the child now drinks without any problems [Traditional healer 1].	The child does drink the medicine. It was just the [LPV/r] that was a problem but then I started mixing it with juice [Caregiver 3].
Micro-	This system involves the child and his/her interactions with all those involved in treatment and care: caregiver, counsellor, nurse, doctor, traditional healer.	Caregiver–child relationship *Grandmothers as caregivers Denial amongst fathers* Characteristics of the caregiver	Yes because in the Zulu culture the man is the head of the house. Sometimes when the father is around when the mother takes the child home with the medication you'll find that the father will refuse the child taking medication […] and say my child is not sick so they shouldn't be getting this ARV medication. [Doctor 3].	When the weight increases the dose must increase so you have to explain to the granny how to give the medication to the child and sometimes it becomes difficult for them because they are very old so the measurements, how to measure properly for the child. And they usually tell that children usually maybe go away for playing and the child doesn't come back if it's time for the medication [Nurse 4].	and these days we know that most of the children – the caregivers are their grannies who do not understand, you know, the measurement [of the treatment doses] and especially with children another thing is that as the children gain weight, the dosage has to change [Counsellor 3]	Like in one of these days one [caregiver] came to me saying that she does not know how to tell the father that the child is positive. I told her to try and tell him because the child is in trouble, he needs to know [Traditional healer 6].	When I first found out that the child has a problem the mother had not told me and it hurt me that my son could have died without knowing anything so I kind of like hated the mother at first because she had not told me but then when I started giving the child the medication, it came to me that since this was her first child, she didn't really know what to do because she was still young so I forgave her because she is a still a child and her first child is sick [Caregiver 3]
Meso-	This system involves the interaction between all the members of the micro system. For example Caregiver–counsellor relationship.	Caregiver–health care worker interaction *Language barriers Multiple caregivers*	We try to find out what's happening and you try to you know repeat a question or ask them do you really understand and sometimes they'll say no and you ask the nurses to translate again. Obviously I don't speak any Zulu, I also don't understand any Zulu so whether the translation is correct I also don't know[Doctor 5].	You find that some children their mothers died long ago so they are brought by different people. This month you see this one, next month you see that one so that becomes a problem because what you have talked with this person next month the child is brought by another who doesn't know [Nurse 4].	Even when you tell her – just say ma'am I'm only there for this week and I won't be there the following week. Even if you want to give this person like the proper information but it's useless because it's pointless because this person is not going to be there in a few months down the line you know [Counsellor 10].	Mostly the children are left to be raised by the grannies and they [the mothers] do not make them aware that the children are positive [Traditional healer 2].	Well I have never had a problem with the clinic, actually speaking, those people who work there motivate you and they advise you, some people get offended but at the end they realize that they are getting good advice at the clinic [Caregiver 2].
Exo-	This system involves the influence of the family network and clinic context.	Experience at the clinic *Adherence counselling and training*	It's all very well we find out the information, but you then have to have … again equip the counsellors with the skills to …' how do you address a case if you hear or A you hear B or C: this is what you might do or you might discuss with the patient [Doctor 1].	This must process [adherence counselling] must be done continuously. It mustn't be once [Nurse 4].	You know, so it's – it's because of I mean I think there should be like a constant like educational sessions in terms of how they should continue, you know taking their treatment [Counsellor 2].		Me for myself, I am also positive and I think 2 classes are enough because I already know about HIV. I didn't know how to drink the medication and that is what I wanted to know so 2 classes for me are enough [Caregiver 3].
Macro-	This system involves the influence of poverty and culture.	Poverty *Food insecurity* Traditional medicines	So last week I had a lady at the clinic and child had a raised viral load, couldn't get to the bottom of it, … turns out they tend to run out of money from about the 20th of each month so basically for about a third of the month, the child, …, refuses to take medicine if there is no food or hardly any food [Doctor 2].	They said this child is eating more if they're taking this treatment so at home there's no food [Nurses 3].	And there are some traditional healers that they tell the people that if you use my medication you will be cured … [Counsellor 5].	Yes we do give them medication but we ask the care giver on the times when they give them the ARVs and allow 1h30 min for them to take the traditional medication so not to interrupt the process of the ARV's working on the child's system [Traditional healer 6].	Well I have never been to a traditional healer because when the child first started to get sick her body temperature was very high so I just took her to the clinic … [Caregiver 5].

#### Individual-level: medication difficulties

Doctors, nurses, counsellors and traditional healers were unanimous about the difficulty that children had with the palatability of Lopinavir/ritonavir. Caregivers indicated that mixing medicines with a sweetener such as a liquid multivitamin, juice or peanut butter improved the taste for children.

#### The micro-system: caregiver–child relationship and characteristics of the caregiver

Caregivers stated that their failure to disclose the child's status to household members disrupted their ability to administer medication. For example, respondents indicated that in some households the father refused to accept that his child was HIV-infected, which would force caregivers to hide the child's medication.

#### The meso-system: caregiver–health worker interaction

Doctors, nurses and counsellors stated that language barriers created difficulties in communicating with caregivers about difficulties regarding medication administration. Although nurses and counsellors were able to communicate with caregivers in isiZulu, they reported that caregivers struggled to understand important information about their treatment such as changes in dose amounts. Further, although grandmothers were the primary caregiver, other household members also administered medication to the child, which became problematic when doses were changed. This problem was exacerbated when communication among these family members was poor.

#### Exo-system: adherence counselling and training

Doctors stated that the skill level of adherence counsellors was generally low, reflecting the poor quality of the training they received. They stated that counsellors were neglected in their role and received no debriefing, minimal supervision, and once-off training sessions that were in some instances poorly conducted. Regarding the pre-ART HIV sessions that caregivers received, doctors and counsellors agreed that caregivers were given too much information over a short period of time. They stated that it was emotionally and cognitively burdensome for a caregiver to accept the child's HIV diagnosis, receive counselling within two days and be expected to commence treatment immediately. Surprisingly, caregivers felt that the sessions were adequate, and that they were comfortable asking for help when needed.

#### Macro-system: food insecurity and traditional medicines

All the caregivers participating in the FG were unemployed, and some received a government grant (see Table 2). Respondents stated that a healthy child threatened the financial stability of many households, as an improvement in health also meant a greater appetite, thus requiring more food for the household. Only one caregiver in the FG had consulted with a traditional healer about difficulties with treatment. Traditional healers however stated that they were consulted by caregivers but that it was difficult to engage with them about medication adherence as caregivers were reluctant to disclose the child's HIV positive status.

## Discussion

Our study is unique in its representation of each of the members involved in the treatment and care of a child on ART in this context. By including each of these role players we were able to demonstrate the relative influence of the micro-, meso-, exo- and macro-systems on paediatric adherence to ART. Vreeman et al. (2009) demonstrated that paediatric adherence among Kenyan children is best understood as behaviours that are shaped by the context in which adherence occurs, thus moving beyond just the individual and caregiver-related factors (Vreeman et al., 2009).

Despite being largely confirmatory, our results indicate that children struggle with unpalatable syrups such as LPV/r (Haberer & Mellins, 2009) which causes vomiting and disrupts dosing when the syrup is not readministered after vomiting. Caregivers are forced to disguise the bitter tasting medication with sweet alternatives, which may be difficult in households where food is scarce.

On a micro-level, grandmothers were considered primary caregivers when a biological mother had passed away or had migrated for work. Most caregivers reported good relationships with the children under their care. However, relationship between each of the other respondents and the caregiver was complicated, especially in cases in which more than one caregiver cared for an individual child and rotated clinic visits among themselves. Clinic staff reported that inconsistent caregivers attending clinic visits made consultations difficult and expressed concern about medication responsibilities being shared at a household level, as miscommunication among caregivers could lead to incorrect medication dosing.

Similar to other findings (Haberer & Mellins, 2009), grandmothers were frequently unable to fulfil their responsibilities as they could not understand the treatment regimen, a problem compounded by the fact that they were caregivers to many children. Poor comprehension of the treatment regimen was also due to exo-level factors such as poor training of adherence counsellors and rushed adherence counselling. A recent systematic review effectively demonstrated the poor level of training and debriefing of counsellors in South Africa, and their subsequent experience of being unappreciated and excluded from the medical hierarchy (Petersen, Fairall, Egbe, & Bhana, 2014).

On a macro-level, food insecurity was a major barrier to caregivers fulfilling their responsibilities, and was worse in cases in which caregivers were responsible for many children. A recent study from KwaZulu-Natal indicated that rural households survive on less than ZAR12 (I.I4USD) per day (D'Haese et al., 2013).

## Conclusions

Caregiver consistency is important and may take the form of regular monitoring of the person attending clinic, monitoring regular communication between caregivers and ensuring that treatment-related recommendations are effectively passed on to the person responsible. Finally, we recommend adequate training of adherence counsellors with regular supervision, training and debriefing.

## Strengths and limitations

The study provides a broad range of perspectives expressed by the key role players in the treatment and care of children on ART. Due to the small sample size and nature of the data, the findings cannot be generalised, but may be used to inform future research.
